# Non-Tuberculous Mycobacteria: Single Center Analyses of Risk Factors, Management and Mortality Outcomes of Adults with HIV

**DOI:** 10.3390/diagnostics14232682

**Published:** 2024-11-27

**Authors:** Lamla Nqwata, Jotam G. Pasipanodya, Marianne Black, Charles Feldman

**Affiliations:** 1 Department of Internal Medicine, University of Witwatersrand, Johannesburg 2193, South Africa; 2 Department of Medicine, Vanderbilt University Medical Center, Nashville, TN 37232, USA

**Keywords:** non-tuberculous mycobacteria, long-term outcomes, Sub-Saharan Africa, *Mycobacterium avium* complex, *Mycobacterium abscessus* complex, *Mycobacterium kansasii*

## Abstract

Background/Objectives: In sub-Saharan Africa, there is paucity of data regarding non-tuberculous mycobacterial (NTM) infections, leading to underappreciation of disease burden. Consequently, fewer resources are allocated, leading to potential adverse outcomes. This study examines long-term mortality and risk factors of South African patients with positive NTM samples. Methods: We conducted a retrospective analysis of clinical isolates of NTMs between 1 January 2010 and 30 June 2017. We retrieved and thoroughly reviewed the corresponding medical records of patients treated at Charlotte Maxeke Johannesburg Academic Hospital. Outcomes were compared between patients who underwent different therapy regimens, including macrolide-based regimens and ‘watchful waiting’. Results: A total of 123 patients were followed for a median of 1 year (interquartile range [IQR], 0.5–4.5). The median age was 39 years (IQR, 31–51) with male predominance, 58%. The common comorbid conditions were HIV (encountered in 78%) and previous TB (58%). Pulmonary disease due to *Mycobacterium avium* complex (MAC-PD) was found in 74% of patients, *M. fortiutum* in 5%, and *M. gordonae* in 4%. The mortality relative risk for patients on initial macrolide-containing therapy was 0.54 (95% confidence interval [CI], 0.22–1.36), *p* = 0.194, while that for macrolide-free antimicrobials was 1.38 (95% CI, 0.57–3.34), *p* = 0.471. The adjusted hazard rate for mortality with low CD4 counts < 50 cells/mm^3^ was 2.79 (95%, 1.20–6.50), while that for unknown CD4 counts was 4.01 (95% CI, 1.17–13.77), compared to CD4 counts > 50 cells/mm^3^. Conclusions: Among HIV patients, NTM-PD predominated, and not disseminated disease. MAC-PD was the most common infection. Low CD4 counts was a significant risk factor for early death, while sex, NTM species, macrolide therapy, and previous TB were not.

## 1. Introduction

Non-tuberculous mycobacteria (NTM) represents a diverse and large group of ~200 mycobacteria organisms that do not belong to the *Mycobacterium tuberculosis* complex (MtbC) [1], hence the monikers *Mycobacteria* other than tuberculosis or atypical *Mycobacteria* [2,3,4]. Most NTM species are environmental organisms, ubiquitous in soils, household water, and dust [5,6], and when isolated in the human host, are usually considered either an opportunistic infection or nonpathogenic colonizers. Consequently, diagnosing NTM disease is both challenging and costly [3,4], which contributes to NTM infections being frequently non-notifiable in many settings. The American Thoracic Society (ATS) and Infectious Diseases Society of America (IDSA) even recommend complicated strict criteria for diagnosis and another criteria for initiating antimicrobial therapy [7,8].

Unfortunately, this results in an under-appreciation of the overall NTM disease burden, so much that fewer resources are allocated for proper NTM diagnosis, treatment, and public health control [9,10,11]. Meanwhile, the global NTM prevalence is increasing [7,8], driven by relatively higher incidence of pulmonary *Mycobacterium avium* complex (MAC) among HIV-negative elderly female patients mostly from resource-replete settings with low tuberculosis (TB) prevalence [3]. Recent meta-analyses and population studies performed in these high-resource settings revealed variable NTM disease risk factors and all-cause mortality rates ranging between 19% and 77%, with most deaths recorded during therapy for NTM pulmonary diseases (NTM-PD) [12,13,14,15,16]. Here, we hypothesized that in resource-limited settings with high TB and HIV prevalence, like South Africa, NTM disease risk factors and overall mortality rates may differ from those observed in high-resource, low TB prevalence areas [1,8,11]. Resource-constrained settings, like South Africa, have low healthcare budgets, and even much lower resources are allocated to rarer diseases like NTMs. Therefore, our primary study objective was to describe the NTM distributions as well as to identify risk factors for long-term survival among patients treated at our institution.

NTM infection and disease progression depend on whether the host has an intact immunity. Disseminated NTM disease has mostly been described in patients with compromised immune systems, such as HIV disease with low CD4 counts [17,18]. However, localized NTM-PD and extra-pulmonary disease, like cervical lymph nodes which is more common in children < 5 years old [19], has been mostly reported in patients with an intact immunity [3,4]. Studies describing the natural trajectory and outcomes of NTM diseases in HIV patients are mostly from resource-replete settings [20,21,22]. Those few studies from resource deplete settings, like Kenya and Uganda, were reported at the height of the AIDS pandemic. Importantly, the studies did not identify disseminated NTM among their AIDS patients [23,24]. Here, we describe long-term mortality of patients from whom NTM organisms were recovered from clinical samples of patients with concurrent HIV infection. We also determined whether initial severe NTM disease, like disseminated NTM or high bacterial load, associated with poorer long-term survival.

ATS/IDSA recommends at least three-drug combination therapy that includes a macrolide for *Mycobacterium abscessus* complex (MABSC), *Mycobacterium kansasii*, and MAC. These are the most frequently encountered NTM organisms responsible for ~95% of NTM-PD worldwide [3,4,8]. Previously, the 2007 ATS/IDSA guidelines [4] recommended ‘watchful waiting’ instead of aggressive initiation of chemotherapy for patients with indolent or nonprogressive NTM-PD, because the adverse effects from drugs were considered worse than NTM disease effects. In resource depleted settings, some NTM-PD patients are inadvertently started on empiric anti-TB drugs based on positive acid-fast stain microscopy results, while others are started on ATS/IDSA-recommended macrolide-containing regimens or ‘watchful-waiting’, as was standard practice prior to the 2020 ATS/IDSA guidelines [25,26]. This variation of prescribed antimicrobials allows for retrospective assessment of the impact of different NTM regimens on patient well-being.

All-cause mortality is a direct measure of patient well-being, unlike sputum culture conversion (SCC) at 6- and 12-month time points, which are the clinical endpoints recently used to assess the efficacy of antimicrobial interventions against NTM infections [27,28]. In one study [29], previous TB disease provided significant protection of 48% (95% confidence interval [CI], 0.36–0.76) against MAC, suggesting acquired broad antimycobacterial immunity that persists in the human host after TB disease [17,29,30]. Here, we compared survival across different therapy regimens, including macrolide-based combination, ‘watchful waiting’ against different NTMs species, at 6-month during therapy, at end-of-therapy, and after long-term post-therapy follow-up in patients with and without previous TB.

Our study of 123 patients recruited from the Charlotte Maxeke Johannesburg Academic Hospital (CMJAH), of whom 96 (78%) had concurrent HIV, revealed that majority 90 (74%) of these patients had localized MAC-PD and not disseminated disease, despite low baseline CD4 counts. Importantly, compared to “watchful waiting”, both macrolide-based and macrolide-free regimens did not provide significant survival benefits, even among NTM patients with previous TB disease.

## 2. Materials and Methods

### 2.1. Setting and Study Design

We performed a retrospective cohort study with examination of medical charts, radiologic records, and the vital statistics records of patients from whom NTM organisms were isolated, in the National Health Laboratory Services (NHLS) database. Next, the medical records at CMJAH in Gauteng province of South Africa for the period between 1 January 2010 and 30 June 2017 were used to identify additional eligible patients with NTM-PD or disseminated NTM disease. One of the authors (LN) retrieved data on positive NTM isolates, and reviewed medical charts and all clinical information following a previously established study protocol.

CMJAH, with a compliment of >4000 professional staff and 1088 inpatient beds, serves as the main teaching hospital for the University of Witwatersrand. CMJAH also provides referral services for Gauteng province and other neighboring northern provinces of South Africa.

### 2.2. Inclusion and Exclusion Criteria

All adult patients (≥18 years of age) with clinical samples that had a documented positive culture result with an NTM isolate were included in this study, while those with insufficient records or results considered contaminants were excluded. The medical records associated with each positive NTM isolate were retrieved, and data extracted onto standardized electronic spreadsheets. The following demographic and clinical data were abstracted and reviewed: age, sex, serial weight, height, body mass index, smoking, other comorbid conditions, radiographic findings (chest Xray [CXR] and computed tomography [CT] scan reports), microbiologic findings (serial sputum acid-fast bacilli smear grade [AFB], time-to-positive [TTP] from MGIT cultures), HIV test results, and the initial antimicrobial therapy regimens. Patients with a diagnosis of clinical or culture-confirmed *Mtb*C within the preceding year of NTM isolation were identified as concurrent *Mtb*, while those with more remote history and documented prior TB treatment were designated previous TB. NHLS used two liquid culture media, the Mycobacteria Growth Indicator Tube (MGIT 960) system and the BACTEC 460 TB system, to grow the NTMs from clinical samples. To identify NTMs from culture, a Genotype-Mycobacterium common mycobacteria was used for the most common clinically significant species, and Genotype Mycobacterium additional species for additional less common NTM species.

### 2.3. Outcomes Assessment

Gauteng provincial electronic vital records, Gauteng TB registers, and patients’ medical charts from CMJAH were used to ascertain the survival of each patient at end of study, as well as to obtain the death dates of deceased patients. Time zero was defined by the date of first isolation of NTM. The minimum therapy duration for most NTM diseases is 24 months [3,4]; therefore, deaths within 24 months after NTM isolation were considered death during therapy, especially if patients had records of receiving antimicrobial therapy. Successful treatment outcomes were called on patients who were alive at end of study. Disseminated NTM infection was defined by recovery of organisms from blood, bone marrow, or liver biopsy specimens, while localized infection or disease were ascribed if the organisms were only recovered at those other sites. Since initial bacterial load has been reported to predict both NTM disease progression and outcomes [31,32], we computed initial mycobacterial burden in log_10_ colony forming units (CFUs), from TTP values (in days), using previously described methods [33].

### 2.4. Antimicrobial Treatment

First, initial treatments were characterized into receipt of some antimicrobials versus none. Thus, patients on ‘watchful waiting’ or those who were not on initial antimicrobials after reviewing their medical charts were assigned to the no antimicrobial group [4]. In resource-limited settings, patients with positive smear sputum microscopy and related clinical symptoms are usually started on TB therapy, in accordance with standard World Health Organization (WHO) guidelines [11,25,26]. Next, the initial antimicrobials received were stratified by macrolide-containing (clarithromycin or azithromycin) versus macrolide-free regimen (i.e., anti-TB regimens without clarithromycin or azithromycin). MAC therapy was defined as an initial regimen of macrolide plus ethambutol and rifamycin in accordance with ATS/IDSA guidelines [3,4].

### 2.5. Statistical Analysis

Mean and median values and corresponding measures of spread were used to compare the distribution of continuous variables, while frequencies and percent values were used to assess the distribution of categorical variables. We estimated absolute risk reduction in mortality by comparing the different NTM regimens observed. The number needed to treat (NNT) was computed as the reciprocal of the absolute risk reduction, while Fisher exact tests were used to compare the proportions. A *p*-value < 0.05 was considered statistically significant; all tests were two-tailed.

Log-rank tests were used to compared Kaplan–Meier curves, while backward multivariate Cox regression analyses were employed to compute the adjusted hazard ratio (aHR) and the corresponding 95% confidence interval (95% CI). We tested a priori a multivariate model comprising age, sex, CD4 counts, HIV test, previous TB, NTM species, and antimicrobials. The analyses and graphics were performed using GraphPad (GraphPad, San Diego, CA, USA), R, and STATA version 16, (STATA Corp, College Station, TX, USA) software.

## 3. Results

### 3.1. Baseline Patient Characteristics:

Of the 199 positive NTM results retrieved from NHLS, 123 (62%) met both the inclusion and exclusion criteria and were selected for further analyses. Of these, only three (2%) NTM positive samples were obtained from blood, bone marrow, or lymph nodes, sites commonly associated with disseminated disease. Among the 76 excluded NTM isolates, 23 (12%) were recovered from pediatric patients and 53 (27%) were contaminants or did not have corresponding medical records.

Table 1 reveals male predominance, 71 (58%), and a relatively young cohort with a median age of 39 (IQR, 31–48) years. One third (40) of all patients had previous TB disease, with bronchiectasis and/or cavitation documented in only 16 (13%) patients. Most patients (96 [78%]), had a positive HIV test result, pancytopenia, and were severely immunosuppressed; however, only 40 (42%) patients had baseline CD4 counts of ≤50 cells/mL^3^. Patients with previous TB were significantly more likely to have CXR abnormalities, including cavitation and bronchiectasis (*p* < 0.005).

### 3.2. Prevalent Non-Tuberculous Mycobacteria Species

**Figure 1:**
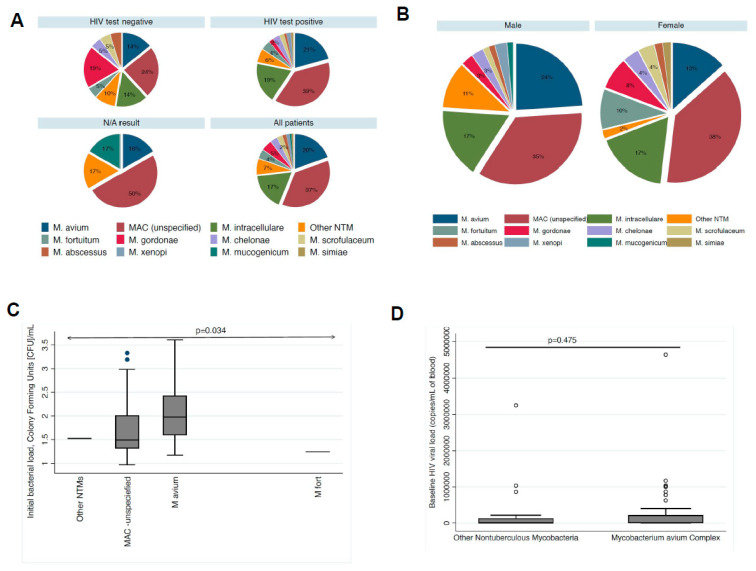
Non-tuberculous Mycobacteria (NTM) distribution, initial bacterial load, and association with patients’ HIV viral load at baseline. The distribution of the different NTM species and *Mycobacterium avium* subspecies are stratified by HIV test result in (**A**). There were 20 (16%) *M. intracellulare* isolates, 45 (37%) *M. avium* isolates, and 24 (20%) unspecified *M. avium Complex* (MAC) species among all NTMs. There were 10 (8%) other NTM isolates that were not speciated. Only two (1.62%) isolates were identified as *Mycobacterium abscessus.* (**B**) The distribution of the different NTMs between sex was significantly different, *p* = 0.037, with all six *M. fortuitum* isolates identified recovered from female patients, while all six non-speciated NTMs recovered from male patients. However, 54/71 (76%) of male patients and 36/52 (69%) of female patients had MAC species, *p* = 0.399, suggesting that the distribution of pulmonary MAC did not significantly differ in this population. (**C**,**D**) Here, the distribution of baseline NTM bacterial load and HIV viral load is compared, respectively, between NTM species. As shown, the initial bacterial load was significantly higher with MAC isolates than non-MAC isolates. Similarly, there was no significant difference in baseline HIV viral loads between patients with MAC versus non-MAC infections. The median HIV viral load was 6950 (range 40–218,000) copies per milliliter (mL) of blood at baseline.

For ease of comparison with other studies in the literature, we enumerated the distribution of >10 different NTM species in the entire cohort and stratified by HIV and sex (Figure 1). Two or more isolates in the same patients, i.e., as ‘mixed’ NTM infections, were observed in 10 (8%) patients. The mean load for *M. avium* was 2.07 cfu/mL versus 1.25 cfu/mL for *M. fortuitum* (*p* = 0.034).

### 3.3. Follow-Up and Overall Mortality

**Figure 2:**
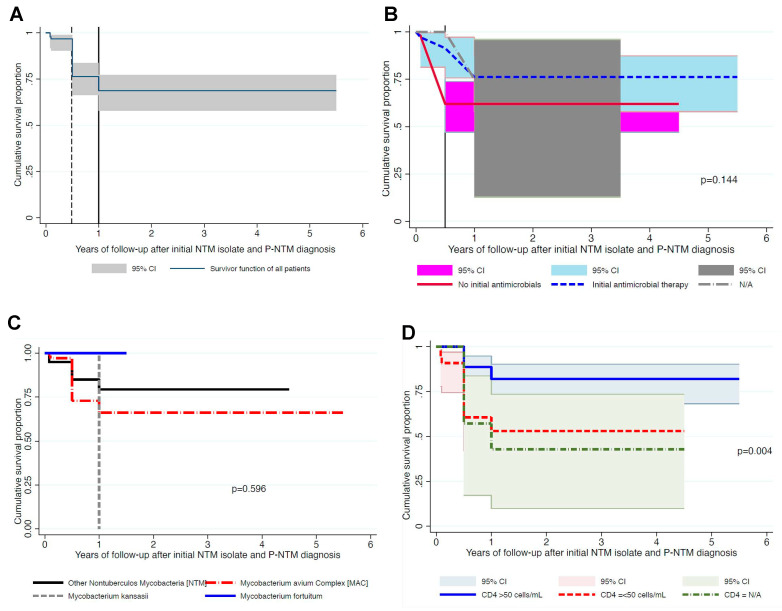
Overall cumulative survival proportions of patients with non-tuberculous mycobacteria and the impact of initial antimicrobial therapy, baseline CD4 counts, and different non-tuberculous mycobacteria (NTM) species on survival. (**A**) Overall survival with the vertical lines (dotted line at 6 months and full line at 12 months) depicting survival at different times along therapy. (**B**) Survival stratified by receipt of antimicrobials. Although there was no statistical significance across the entire follow-up, when comparisons were restricted to survival at 6 months (indicated by full vertical line), survival was significantly higher in patients who received initial antimicrobial agents. (**C**) No significant difference when patients were stratified by NTM species, while (**D**) risk of death was higher in those with low CD4 counts or missing CD4 data.

The 96 patients who contributed 28 deaths were followed-up for a total of 168.26 person-years, ranging from 0.08 to 5 years. The median and mean follow-up rates in person-years were 1 and 1.81, respectively. Those 28 deaths contributed an overall mortality incidence of 16.7 (95% confidence interval [CI], 11.5–24.1) per 100 person-years. Figure 2A shows that 19/28 (79%) of these NTM deaths occurred within 6 months of NTM isolation or during therapy. The cumulative deaths at 0.5, 2, and 5 years were 21%, 34%, and 34%, respectively.

#### 3.3.1. Impact of Initial Antimicrobial Therapy on Mortality

Majority of patients on antimicrobials, 31/63 (49%), were on macrolide-based regimens, as recommended by ATS/IDSA. The drug combinations varied, and included fluoroquinolones, but were given for varying therapy durations (Table 2). There were 60/123 (49%) patients who were on ‘watchful waiting’ without any antimicrobial therapy or had antimicrobial data missing, most likely because of failure to make the NTM diagnosis at baseline. When compared to patients who did not take any antimicrobials, the relative risk for death among patients on macrolide-containing therapy was 0.54 (95% CI, 0.22–1.36), *p* = 0.194, while that for patients on macrolide-free antimicrobials (i.e., without clarithromycin or azithromycin) was 1.38 (95% CI, 0.57–3.34), *p* = 0.471. These findings did not change in the initial prespecified multivariate Cox regression analysis. Based on these findings, we combined all antimicrobials into one group and reran both the univariate and multivariate analyses. The Kaplan–Meier curves are shown in Figure 2B. The combined multivariate analysis results, before backward elimination, are shown in Supplementary Table S1. As shown, the relative risk ratios for patients on antimicrobial agents did not significantly change, even when the analysis was restricted to patients with MAC isolates.

The NNT to prevent one death associated with antimicrobials was 25; however, the 95% confidence interval crossed 0. No NTM patients received any surgical resections in this cohort. Post hoc analyses that accounted for a drop-off rate of 22% revealed that our sample size of 123 patients had power (1 − β) of 0.81 to detect a hazard ratio of 0.28.

#### 3.3.2. Univariate Analysis of Other Factors Associated with Mortality

There was no significant difference in survival observed between the different NTM species, *p* = 0.596 (Figure 2C). Similarly, recurrent TB and previous TB were not associated with mortality, *p* = 0.106 (Supplementary Figure S1) and *p* = 0.144 (Supplementary Figure S2), respectively.

### 3.4. Final Multivariate Analyses

Backward elimination Cox proportional hazard regression analysis, which started with age, sex, NTM species, receipt of antimicrobial therapy, HIV status, and CD4 counts in the initial model, revealed that only CD4 counts significantly predicted mortality (Table 3, Figure 2D). Surprisingly, even though MAC represented 74% of isolates, NTM species did not predict survival. The final adjusted hazard rate for time-to-death for low CD4 counts ≤ 50 cells/mm^3^ of 2.79 (95% CI, 1.20–6.50), while that for those with unknown CD4 counts was 4.01 (95% CI, 1.17–13.77) when compared to CD4 counts > 50 cell/mm^3^.

## 4. Discussion

Treatment-related survival over the first two years was poor, as 21% of all patients died during treatment, representing all treatment-related deaths. Another 22% of patients were not evaluable because of missing data. In comparison, only 21 (12%) out of 172 NTM patients from Oregon, USA, died in a similar two-year observation period [22]. We anticipate that some patients lost to follow-up may have returned to the rural areas where they likely passed away, as has previously been observed among South African pulmonary TB patients who default on follow-up [34]. Regardless, the overall mortality rate of 34% after 5-years follow-up (17 deaths per 100 person-year [95% CI, 12–24]), is not significantly different from the 35.1% reported in a comparable cohort of 368 NTM patients from Oregon [22,35]. This suggests that for the endpoint of 5-year all-cause mortality, the rates are comparably poor across both high- and low-resource settings, potentially due to the suboptimal efficacy of existing NTM therapies. A larger retrospective study from South Korea reported mortality rates of 18% after a median follow-up of 6 years [36]. A recent larger lookback study of 1558 patients reported that MAC was the predominant NTM species (>78%) across all three cohorts.

NTM-PD exhibits variable disease progression and responses to standard therapy regimens, necessitating additional context to explain observed differences across cohorts. The Oregon cohort was older, with a mean age of 65 years, and was predominantly female (53%), with primary causes of death being COPD/emphysema, cancer, and other lung causes in 18% of cases. In contrast, our South African cohort was younger (mean age of 40) and predominantly male (58%), with *M. abscessus* complex infrequently encountered. Causes of death were unavailable for this cohort; however, the early timing of deaths suggests they were likely NTM-related. The South Korean cohort had a median age of 61 years and was 74% female.

We found no significant long-term survival benefits in patients treated with macrolide-containing regimens (clarithromycin or azithromycin) compared to those on macrolide-free antimicrobials or “watchful waiting”. Additionally, previous TB disease did not confer any survival benefit, suggesting that any acquired antimycobacterial immunity may only protect against disseminated MAC rather that MAC-related death [29]. Like previous studies from Kenya and Uganda, we did not find cases of disseminated NTM in our cohort [23,24]. Two non-mutually exclusive explanations for poor therapeutic outcomes include potentially advanced immunosuppression and NTM-PD among our patients, as well as suboptimal drug exposure levels. We hypothesize that suboptimal NTM chemotherapy applies across settings.

Recent pharmacokinetic (PK) and pharmacodynamic (PD) studies using a hollow fiber model of MAC, which mimics patient drug exposures in lesions, show inadequate drug levels for the three main anti-MAC drugs [37,38,39,40,41]. Effective ethambutol concentrations required a Cmax/MIC ratio of 13 for adequate intracellular MAC bacilli killing, which is only achievable with high doses (≥50 mg/kg twice or thrice weekly), when population PK variability is taken into account [37]. However, current ATS/IDSA guidelines recommend much lower doses (15–25 mg/kg), likely leading to suboptimal exposures. Similarly, azithromycin required an AUC/MIC ratio of 7.5, with much lower levels associated with higher acquired drug resistance [42]. Follow-up clinical PK/PD studies confirmed that low macrolide and ethambutol exposures may contribute to poor outcomes, underscoring the need to optimize current treatment regimens as drugs are developed [43].

In our NTM-PD cohort, lung tissue damage and cavitation from prior pulmonary TB was a common comorbidity. Although *M. fortuitum* is sometimes regarded as less virulent [44,45], it is plausible that the six female patients with *M. fortuitum* in our study had post-TB lung damage with NTM-PD rather than mere colonization. It remains unclear whether structural lung destruction per se predisposes patients to NTM-PD disease, or if subsequent treatment for those disorders with antibiotics and inhaled corticosteroids selectively favors certain NTM organisms and not others [46,47]. Recent studies, using both culture and non-culture molecular methods, have found greater NTM species diversity, with some clinical samples containing multiple bacterial species, particularly in patients with nodular bronchiectasis [47,48,49,50,51]. Additional evidence suggests that pulmonary microbiome dysregulation and reduced mycobacterial diversity correlate with more severe lung damage [49]. The causal link between structural lung damage and subsequent NTM-PD in resource-limited settings warrants further investigation.

Our cohort exhibited a co-existence of multiple NTM species in 8% of cases, aligning with findings from US studies. This suggests that NTM disease, superinfection, and colonization may coexist along a spectrum, necessitating a nuanced approach to diagnosis that keeps pace with advancements in molecular technology [47,50,51,52]. The potential contribution of post-NTM lung damage to premature mortality requires further evaluation, similar to studies on post-TB sequelae [53,54].

Or study faced limitations, including some missing baseline and follow-up data. Over one-third of patients lacked chest radiography data, and empiric antimycobacterial treatments sometimes prevented the collection of viable serial sputum samples or drug susceptibility testing. Consequently, we did not apply ATS/IDSA criteria, which can be challenging to implement, even prospectively, in our setting. Finally, as a single center study, these findings may not be generalizable across South Africa or sub-Saharan Africa.

## 5. Conclusions

In this single-center study, cumulative survival probabilities at 0.5, 2, and 5 years were 79%, 66%, and 66%, respectively. Both macrolide-containing and macrolide-free regimens showed no survival benefits. Poorer survival was observed in severely immunosuppressed patients, reiterating the prognostic significance of low CD4 counts and anti-retroviral therapy. Although mixed NTM infections were uncommon, concurrent HIV and *M. tuberculosis* infections were frequent. Previous TB did not confer any survival advantage, in contrast to previous reports. The epidemiology and clinical characteristics of NTM infections in resource-limited settings appear distinct from those in high resources settings. Further, particularly, prospective cohort studies are needed to clarify the dynamics of NTM diseases in settings where other infections like TB and HIV are endemic.

## Figures and Tables

**Table 1:** Baseline demographic, clinical, and laboratory characteristics of study patients.

Variable, Units	Level	All Patients	HIV Test Result			*p*-Value
		*n* = 123 (%)	Negative, *n* = 21 (%)	Positive, *n* = 96 (%)	Unknown, *n* = 6 (%)	
Demographics						
Age, years	Median (IQR)	39 (31–48)	41 (32–58)	38 (31–45)	45 (39–51)	0.329
Weight, KG	Median (IQR)	58 (50–63)	60 (53–68)	57 (49–67)	62 (62–62)	0.528
Sex	Male	71 (58)	8 (38)	59 (61)	4 (67)	0.131
	Female	52 (42)	13 (62)	37 (39)	2 (33)	
Clinical						
Previous TB	None	49 (40)	10 (48)	39 (40)	0	0.001
	Yes	40 (33)	4 (19)	36 (38)	0	
	N/A	34 (28)	7 (33)	21 (22)	6	
Concurrent *Mtb*	None	64 (52)	18 (86)	41 (43)	5 (83)	0.002
	Yes	41 (33)	3 (14)	38 (40)	0	
	N/A	18 (15)	0	17 (17)	1 (17)	
Cavitary CXR	None	17 (14)	1 (5)	16 (17)	0	0.373
	Yes	12 (10)	3 (14)	9 (9)	0	
	N/A	94 (76)	17 (81)	71 (74)	6 (100)	
ATS/IDSA micro	No	91 (74)	18 (86)	68 (71)	5 (83)	0.322
Criteria met	Yes	32 (26)	3 (14)	28 (29)	1 (17)	
Therapy duration, months	Median (IQR)	18 (7–24)	13 (7–21)	18 (8–24)	--	0.653
Laboratory						
HIV viral load, copies/mL	Median (IQR)		--	6950 (40–218,000)	--	
CD4 counts, cells/mm^3^	Median (IQR)		--	65 (19–228)	--	
NTM phenotypes	Rapid growers	14 (11)	3 (14)	10 (10)	1 (17)	0.806
	Slow growers	109 (89)	18 (86)	86 (90)	5 (83)	
NTM species	MAC	90 (73)	11 (52)	75 (78)	4 (67)	0.051
	Other NTMs	33 (27)	10 (48)	21 (22)	2 (33)	
Initial bacterial load (log_10_ CFU)	Median (IQR)	1.60 (0.38–3.56)	1.59 (1.39–1.87)	1.73 (1.39–2.36)	1.49 (1.46–1.66)	0.789

Note: N/A, data not readily available in patient’s medical records; CFU, colony forming unit; CXR, chest X-ray; IQR, inter-quartile range; KG, kilogram; NTM, non-tuberculous mycobacteria; *Mtb*, Mycobacterium tuberculosis.

**Table 2:** Initial treatment strategy and therapy duration by mortality outcomes.

Initial Treatment Strategy	Total
	*n* = 123 (%)
None or “Watchful Waiting”	60 (49)
Macrolide-containing	41 (33)
Macrolide-free (first + second line TB drugs without clarithromycin or azithromycin)	22 (18)
Therapy duration (months)	
Median (IQR)	18 (7–24)
Mean [SD]	15.32 [8.69]
Range	1–26

IQR, interquartile range; SD, standard deviation.

**Table 3:** Frequency and factors associated with time-to-death in univariate and multivariate analysis.

Variable	Level	Frequencies		Univariate Model for All NTMs		Multivariate Model for All NTMs	
		Died, *n* = 28 (%)	Censored, *n* = 68 (%)	Unadjusted HR	*p*-Value	Adjusted HR (CI)	*p*-Value
Sex	Male	16 (57)	43 (63)	Ref--			
	Female	12 (43)	25 (37)	1.25 (0.59–2.65)	0.554		
HIV test	Negative	3 (11)	13 (19)	Ref--			
	Positive	25 (89)	55 (81)	1.79 (0.54–5.95)	0.339		
CD4 counts	>50 cells/mm^3^	9 (32)	45 (66)	Ref--			
	<50 cells/mm^3^	15 (54)	20 (29)	***3.16 (1.38–7.23)***	***0.007***	***2.79 (1.20–6.50)***	***0.017***
	N/A	4 (14)	3 (4)	***3.59 (1.10–11.66)***	***0.034***	***4.01 (1.17–13.77)***	***0.028***
NTM species	Other species	5 (18)	19 (28)	Ref--			
	MAC	23 (82)	49 (72)	1.56 (0.59–4.10)	0.369		
Antimicrobials	None	11 (39)	24 (35)	Ref--		Ref--	
	Some	17 (61)	44 (65)	0.81 (0.38–1.72)	0.576	0.98 (0.83–1.45) *	0.853

* Pooled data; REF denotes the referent category used in analysis; N/A, data unavailable; Figures in bold and italics were significant at 5% alpha level.

## Data Availability

The data presented in this study are available on reasonable request to the corresponding authors.

## Supplementary Materials

The following supporting information can be downloaded at: https://www.mdpi.com/article/10.3390/diagnostics14232682/s1, Figure S1: Comparison of overall survival stratified by concurrent TB; Figure S2: Comparison of overall survival stratified by previous TB; Table S1: Initial Multivariate Cox regression models for all NTM species and for MAC isolates.

## References

[1] Nqwata L., Ouedrago A.R. (2022). Non-tuberculous mycobacteria pulmonary disease: A review of trends, risk factors, diagnosis and management. Afr. J. Thorac. Crit. Care Med..

[2] Yan M., Brode S.K., Marras T.K. (2023). The Other Nontuberculous Mycobacteria: Clinical Aspects of Lung Disease Caused by Less Common Slowly Growing Nontuberculous Mycobacteria Species. Chest.

[3] Daley C.L., Iaccarino J.M., Lange C., Cambau E., Wallace R.J., Andrejak C., Böttger E.C., Brozek J., Griffith D.E., Guglielmetti L. (2020). Treatment of Nontuberculous Mycobacterial Pulmonary Disease: An Official ATS/ERS/ESCMID/IDSA Clinical Practice Guideline. Clin. Infect. Dis..

[4] Griffith D.E., Aksamit T., Brown-Elliott B.A., Catanzaro A., Daley C., Gordin F., Holland S.M., Horsburgh R., Huitt G., Iademarco M.F. (2007). An official ATS/IDSA statement: Diagnosis, treatment, and prevention of nontuberculous mycobacterial diseases. Am. J. Respir. Crit. Care Med..

[5] Biet F., Boschiroli M.L., Thorel M.F., Guilloteau L.A. (2005). Zoonotic aspects of Mycobacterium bovis and Mycobacterium avium-intracellulare complex (MAC). Vet. Res..

[6] Wallace R.J., Iakhiaeva E., Williams M.D., Brown-Elliott B.A., Vasireddy S., Vasireddy R., Lande L., Peterson D.D., Sawicki J., Kwait R. (2013). Absence of Mycobacterium intracellulare and presence of Mycobacterium chimaera in household water and biofilm samples of patients in the United States with Mycobacterium avium complex respiratory disease. J. Clin. Microbiol..

[7] Vinnard C., Longworth S., Mezochow A., Patrawalla A., Kreiswirth B.N., Hamilton K. (2016). Deaths Related to Nontuberculous Mycobacterial Infections in the United States, 1999–2014. Ann. Am. Thorac. Soc..

[8] Okoi C., Anderson S.T.B., Antonio M., Mulwa S.N., Gehre F., Adetifa I.M.O. (2017). Non-tuberculous Mycobacteria isolated from Pulmonary samples in sub-Saharan Africa—A Systematic Review and Meta Analyses. Sci. Rep..

[9] Abate G., Stapleton J.T., Rouphael N., Creech B., Stout J.E., El Sahly H.M., Jackson L., Leyva F.J., Tomashek K.M., Tibbals M. (2021). Variability in the Management of Adults With Pulmonary Nontuberculous Mycobacterial Disease. Clin. Infect. Dis..

[10] Adjemian J., Prevots D.R., Gallagher J., Heap K., Gupta R., Griffith D. (2014). Lack of adherence to evidence-based treatment guidelines for nontuberculous mycobacterial lung disease. Ann. Am. Thorac. Soc..

[11] Agizew T., Basotli J., Alexander H., Boyd R., Letsibogo G., Auld A., Nyirenda S., Tedla Z., Mathoma A., Mathebula U. (2017). Higher-than-expected prevalence of non-tuberculous mycobacteria in HIV setting in Botswana: Implications for diagnostic algorithms using Xpert MTB/RIF assay. PLoS ONE.

[12] Pasipanodya J.G., Ogbonna D., Deshpande D., Srivastava S., Gumbo T. (2017). Meta-analyses and the evidence base for microbial outcomes in the treatment of pulmonary Mycobacterium avium-intracellulare complex disease. J. Antimicrob. Chemother..

[13] Pasipanodya J.G., Ogbonna D., Ferro B.E., Magombedze G., Srivastava S., Deshpande D., Gumbo T. (2017). Systematic Review and Meta-analyses of the Effect of Chemotherapy on Pulmonary Mycobacterium abscessus Outcomes and Disease Recurrence. Antimicrob. Agents Chemother..

[14] Wetzstein N., Kohl T.A., Diricks M., Mas-Peiro S., Holubec T., Kessel J., Graf C., Koch B., Herrmann E., Vehreschild M. (2023). Clinical characteristics and outcome of Mycobacterium chimaera infections after cardiac surgery: Systematic review and meta-analysis of 180 heater-cooler unit-associated cases. Clin. Microbiol. Infect..

[15] Brode S.K., Chung H., Campitelli M.A., Kwong J.C., Sutradhar R., Marchand-Austin A., Winthrop K.L., Jamieson F.B., Marras T.K. (2020). The impact of different antibiotic treatment regimens on mortality in Mycobacterium avium complex pulmonary disease: A population-based cohort study. Eur. Respir. J..

[16] Lee H., Myung W., Lee E.M., Kim H., Jhun B.W. (2021). Mortality and Prognostic Factors of Nontuberculous Mycobacterial Infection in Korea: A Population-based Comparative Study. Clin. Infect. Dis..

[17] Nightingale S.D., Byrd L.T., Southern P.M., Jockusch J.D., Cal S.X., Wynne B.A. (1992). Incidence of Mycobacterium avium-intracellulare complex bacteremia in human immunodeficiency virus-positive patients. J. Infect. Dis..

[18] Chaisson R.E., Moore R.D., Richman D.D., Keruly J., Creagh T. (1992). Incidence and natural history of Mycobacterium avium-complex infections in patients with advanced human immunodeficiency virus disease treated with zidovudine. The Zidovudine Epidemiology Study Group. Am. Rev. Respir. Dis..

[19] Harris K.A., Kenna D.T., Blauwendraat C., Hartley J.C., Turton J.F., Aurora P., Dixon G.L. (2012). Molecular fingerprinting of Mycobacterium abscessus strains in a cohort of pediatric cystic fibrosis patients. J. Clin. Microbiol..

[20] Moore R.D., Chaisson R.E. (1996). Natural history of opportunistic disease in an HIV-infected urban clinical cohort. Ann. Intern. Med..

[21] Benson C.A., Williams P.L., Currier J.S., Holland F., Mahon L.F., MacGregor R.R., Inderlied C.B., Flexner C., Neidig J., Chaisson R. (2003). A prospective, randomized trial examining the efficacy and safety of clarithromycin in combination with ethambutol, rifabutin, or both for the treatment of disseminated Mycobacterium avium complex disease in persons with acquired immunodeficiency syndrome. Clin. Infect. Dis..

[22] Henkle E., Novosad S.A., Shafer S., Hedberg K., Siegel S.A.R., Ku J., Varley C., Prevots D.R., Marras T.K., Winthrop K.L. (2017). Long-Term Outcomes in a Population-based Cohort with Respiratory Nontuberculous Mycobacteria Isolation. Ann. Am. Thorac. Soc..

[23] Gilks C.F., Brindle R.J., Mwachari C., Batchelor B., Bwayo J., Kimari J., Arbeit R.D., von Reyn C.F. (1995). Disseminated Mycobacterium avium infection among HIV-infected patients in Kenya. J. Acquir. Immune Defic. Syndr. Hum. Retrovirol..

[24] Okello D.O., Sewankambo N., Goodgame R., Aisu T.O., Kwezi M., Morrissey A., Ellner J.J. (1990). Absence of bacteremia with Mycobacterium avium-intracellulare in Ugandan patients with AIDS. J. Infect. Dis..

[25] World Health Organization (2010). Treatment of Tuberculosis: Guidelines.

[26] Mbeha B., Mine M., Motswaledi M.S., Dewar J. (2019). Nontuberculous Mycobacteria, Botswana, 2011-2014. Emerg. Infect. Dis..

[27] Griffith D.E., Eagle G., Thomson R., Aksamit T.R., Hasegawa N., Morimoto K., Addrizzo-Harris D.J., O’Donnell A.E., Marras T.K., Flume P.A. (2018). Amikacin Liposome Inhalation Suspension for Treatment-Refractory Lung Disease Caused by Mycobacterium avium Complex (CONVERT). A Prospective, Open-Label, Randomized Study. Am. J. Respir. Crit. Care Med..

[28] Griffith D.E., Adjemian J., Brown-Elliott B.A., Philley J.V., Prevots D.R., Gaston C., Olivier K.N., Wallace R.J. (2015). Semi-quantitative Culture Analysis During Therapy for Mycobacterium avium Complex (MAC) Lung Disease. Am. J. Respir. Crit. Care Med..

[29] Horsburgh C.R., Hanson D.L., Jones J.L., Thompson S.E. (1996). Protection from Mycobacterium avium complex disease in human immunodeficiency virus-infected persons with a history of tuberculosis. J. Infect. Dis..

[30] von Reyn C.F., Barber T.W., Arbeit R.D., Sox C.H., O’Connor G.T., Brindle R.J., Gilks C.F., Hakkarainen K., Ranki A., Bartholomew C. (1993). Evidence of previous infection with Mycobacterium avium-Mycobacterium intracellulare complex among healthy subjects: An international study of dominant mycobacterial skin test reactions. J. Infect. Dis..

[31] Jhun B.W., Moon S.M., Jeon K., Kwon O.J., Yoo H., Carriere K.C., Huh H.J., Lee N.Y., Shin S.J., Daley C.L. (2019). Prognostic factors associated with long-term mortality in 1445 patients with nontuberculous mycobacterial pulmonary disease: A 15-year follow-up study. Eur. Respir. J..

[32] Pan S.W., Shu C.C., Feng J.Y., Wang J.Y., Chan Y.J., Yu C.J., Su W.J. (2017). Microbiological Persistence in Patients With Mycobacterium avium Complex Lung Disease: The Predictors and the Impact on Radiographic Progression. Clin. Infect. Dis..

[33] Ruth M.M., Magombedze G., Gumbo T., Bendet P., Sangen J.J.N., Zweijpfenning S., Hoefsloot W., Pennings L., Koeken V.A.C.M., Wertheim H.F.L. (2019). Minocycline treatment for pulmonary Mycobacterium avium complex disease based on pharmacokinetics/pharmacodynamics and Bayesian framework mathematical models. J. Antimicrob. Chemother..

[34] van Halsema C.L., Fielding K.L., Chihota V.N., Lewis J.J., Churchyard G.J., Grant A.D. (2012). Trends in drug-resistant tuberculosis in a gold-mining workforce in South Africa, 2002–2008. Int. J. Tuberc. Lung Dis..

[35] Novosad S.A., Henkle E., Schafer S., Hedberg K., Ku J., Siegel S.A.R., Choi D., Slatore C.G., Winthrop K.L. (2017). Mortality after Respiratory Isolation of Nontuberculous Mycobacteria. A Comparison of Patients Who Did and Did Not Meet Disease Criteria. Ann. Am. Thorac. Soc..

[36] Kim H.J., Kwak N., Hong H., Kang N., Im Y., Jhun B.W., Yim J.J. (2021). BACES Score for Predicting Mortality in Nontuberculous Mycobacterial Pulmonary Disease. Am. J. Respir. Crit. Care Med..

[37] Deshpande D., Srivastava S., Meek C., Leff R., Gumbo T. (2010). Ethambutol optimal clinical dose and susceptibility breakpoint identification by use of a novel pharmacokinetic-pharmacodynamic model of disseminated intracellular *Mycobacterium avium*. Antimicrob. Agents Chemother..

[38] Ruth M.M., Raaijmakers J., van den Hombergh E., Aarnoutse R., Svensson E.M., Susanto B.O., Simonsson U.S.H., Wertheim H., Hoefsloot W., van Ingen J. (2022). Standard therapy of Mycobacterium avium complex pulmonary disease shows limited efficacy in an open source hollow fibre system that simulates human plasma and epithelial lining fluid pharmacokinetics. Clin. Microbiol. Infect..

[39] Salillas S., Raaijmakers J., Aarnoutse R.E., Svensson E.M., Asouit K., van den Hombergh E., Te Brake L., Stemkens R., Wertheim H.F.L., Hoefsloot W. (2024). Clofazimine as a substitute for rifampicin improves efficacy of Mycobacterium avium pulmonary disease treatment in the hollow-fiber model. Antimicrob. Agents Chemother..

[40] Schildkraut J.A., Raaijmakers J., Aarnoutse R., Hoefsloot W., Wertheim H.F.L., van Ingen J. (2023). The role of rifampicin within the treatment of Mycobacterium avium pulmonary disease. Antimicrob. Agents Chemother..

[41] Boorgula G.D., Jakkula L., Gumbo T., Jung B., Srivastava S. (2021). Comparison of Rifamycins for Efficacy Against Mycobacterium avium Complex and Resistance Emergence in the Hollow Fiber Model System. Front. Pharmacol..

[42] Deshpande D., Pasipanodya J.G., Gumbo T. (2016). Azithromycin Dose to Maximize Efficacy and Suppress Acquired Drug Resistance in Pulmonary Mycobacterium avium Disease. Antimicrob. Agents Chemother..

[43] van Ingen J., Egelund E.F., Levin A., Totten S.E., Boeree M.J., Mouton J.W., Aarnoutse R.E., Heifets L.B., Peloquin C.A., Daley C.L. (2012). The Pharmacokinetics and Pharmacodynamics of Pulmonary Mycobacterium avium Complex Disease Treatment. Am. J. Respir. Crit. Care Med..

[44] Park S., Suh G.Y., Chung M.P., Kim H., Kwon O.J., Lee K.S., Lee N.Y., Koh W.J. (2008). Clinical significance of Mycobacterium fortuitum isolated from respiratory specimens. Respir. Med..

[45] Lee M.R., Yang C.Y., Shu C.C., Lin C.K., Wen Y.F., Lee S.W., Ko J.C., Wang J.Y., Lee L.N., Yu C.J. (2015). Factors associated with subsequent nontuberculous mycobacterial lung disease in patients with a single sputum isolate on initial examination. Clin. Microbiol. Infect..

[46] Brode S.K., Campitelli M.A., Kwong J.C., Lu H., Marchand-Austin A., Gershon A.S., Jamieson F.B., Marras T.K. (2017). The risk of mycobacterial infections associated with inhaled corticosteroid use. Eur. Respir. J..

[47] Cowman S.A., James P., Wilson R., Cookson W.O.C., Moffatt M.F., Loebinger M.R. (2018). Profiling mycobacterial communities in pulmonary nontuberculous mycobacterial disease. PLoS ONE.

[48] Macovei L., McCafferty J., Chen T., Teles F., Hasturk H., Paster B.J., Campos-Neto A. (2015). The hidden ‘mycobacteriome’ of the human healthy oral cavity and upper respiratory tract. J. Oral Microbiol..

[49] Flight W.G., Smith A., Paisey C., Marchesi J.R., Bull M.J., Norville P.J., Mutton K.J., Webb A.K., Bright-Thomas R.J., Jones A.M. (2015). Rapid Detection of Emerging Pathogens and Loss of Microbial Diversity Associated with Severe Lung Disease in Cystic Fibrosis. J. Clin. Microbiol..

[50] Wallace R.J., Zhang Y., Brown B.A., Dawson D., Murphy D.T., Wilson R., Griffith D.E. (1998). Polyclonal Mycobacterium avium complex infections in patients with nodular bronchiectasis. Am. J. Respir. Crit. Care Med..

[51] Griffith D.E., Girard W.M., Wallace R.J. (1993). Clinical features of pulmonary disease caused by rapidly growing mycobacteria. An analysis of 154 patients. Am. Rev. Respir. Dis..

[52] Sulaiman I., Wu B.G., Li Y., Scott A.S., Malecha P., Scaglione B., Wang J., Basavaraj A., Chung S., Bantis K. (2018). Evaluation of the airway microbiome in nontuberculous mycobacteria disease. Eur. Respir. J..

[53] Pasipanodya J.G., Miller T.L., Vecino M., Munguia G., Garmon R., Bae S., Drewyer G., Weis S.E. (2007). Pulmonary impairment after tuberculosis. Chest.

[54] Pasipanodya J.G., McNabb S.J., Hilsenrath P., Bae S., Lykens K., Vecino E., Munguia G., Miller T.L., Drewyer G., Weis S.E. (2010). Pulmonary impairment after tuberculosis and its contribution to TB burden. BMC Public Health.

