# Supplementary material for: Non-Tuberculous Mycobacteria: Single Center Analyses of Risk Factors, Management and Mortality Outcomes of Adults with HIV
Source: Diagnostics (Basel). 2024 Nov 27;14(23):2682. doi: 10.3390/diagnostics14232682 (PMC11640248; doi:10.3390/diagnostics14232682)
Supplement: Supplementary file 1 [file diagnostics-14-02682-s001.zip › diagnostics-3312726-supplementary.pdf]

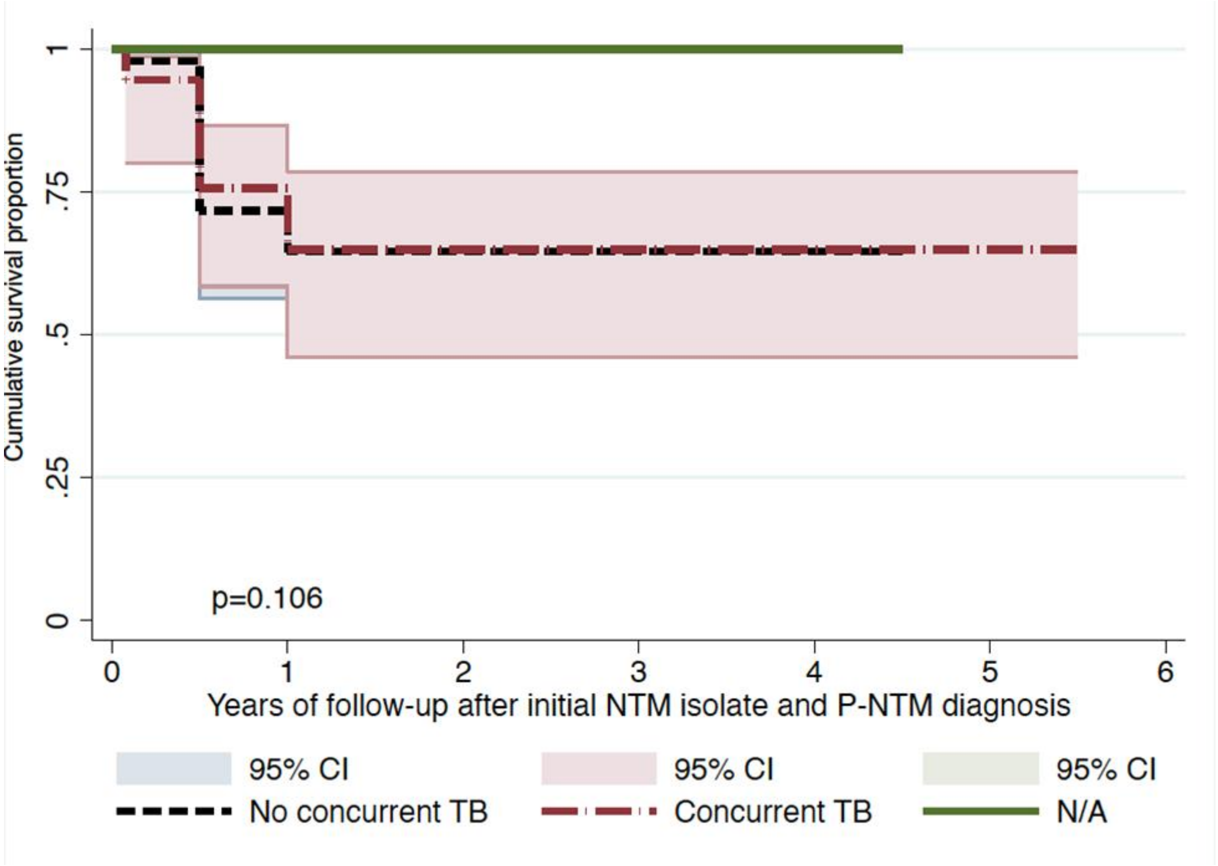

Figure S1. shows comparison of overall survival stratified by concurrent TB.

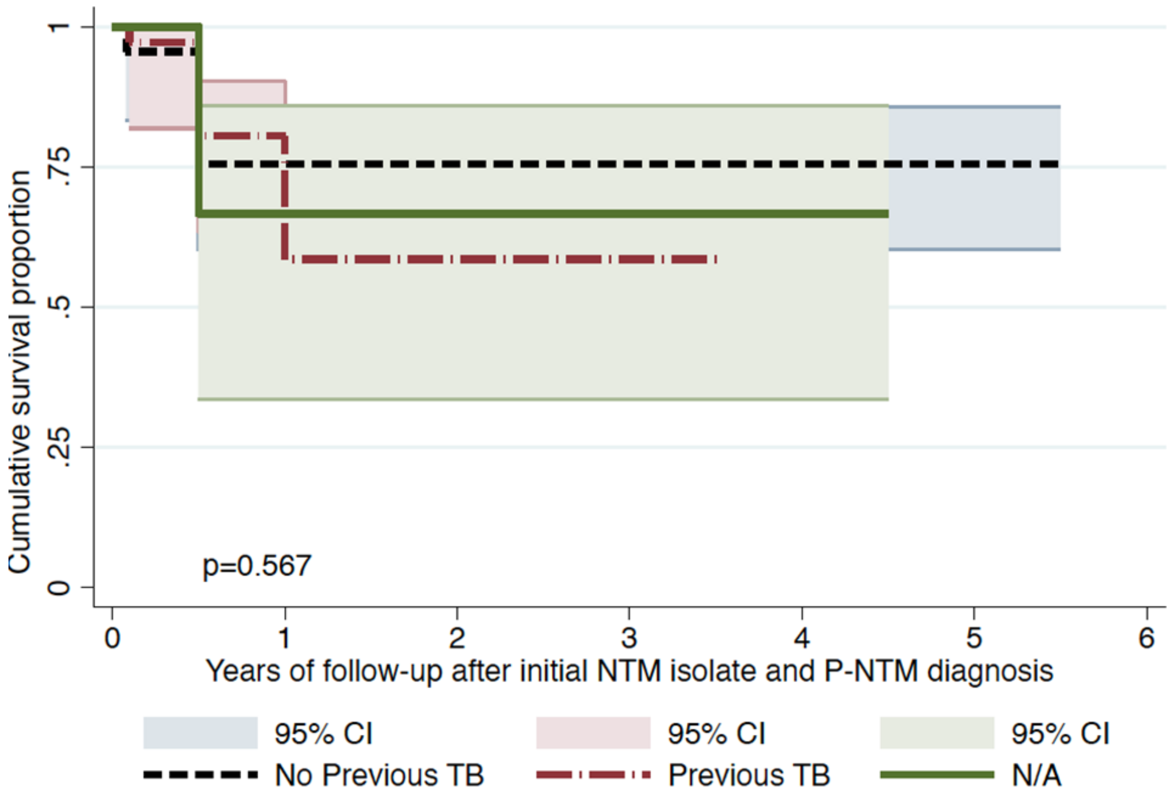

Figure S2. shows comparison of overall survival stratified by previous TB.

Supplementary Table S1. Initial Multivariate Cox regression models for all NTM species and for MAC isolates.

Variable	Level	All NTM Species		MAC isolates only	
		**Hazard ratio (95% CI)**	**P-value**	**Hazard ratio (95% CI)**	**P-value**
**Age**	Continuous age in years	1.01 (0.98 - 1.05)	0.470	1.03 (0.99-1.07)	0.148
**Sex**	Male	Ref--		Ref--	
	Female	1.60 (0.68-3.76)	0.286	1.87 (0.68 – 5.16)	0.224
**HIV test**	Negative	Ref--		Ref--	
	Positive	1.06 (0.25-4.52)	0.937	2.15 (0.23-20.42)	0.506
	N/A	2.67 (0.15-46.51)	0.501	6.20 (0.19-201-78)	0.304
**CD4 counts**	>50 cells/mm^3	Ref--		Ref--	
	<50 cells/mm^3	***3.39 (1.22-9.41)***	***0.019***	2.63 (0.93-7.40)	0.067
	N/A	3.93 (0.88-17.23)	0.074	2.16 (0.47-10.01)	0.324
**NTM species**	Other species	Ref--		N/A	
	MAC	0.78 (0.25	0.671		
**Antimicrobials**	None	Ref--		Ref--	
	Macrolide-containing	0.74 (0.28-1.99)	0.556	0.80 (0.32-1.99)	0.625
	Macrolide-free (First line or Second line TB drugs) without clarithromycin or azithromycin	1.54 (0.58-4.06)	0.386		

REF denotes the referent category used in analysis; N/A, data unavailable; Figures in bold and italics were significant at 5% alpha level.
